# Supplementary material for: Activity-dependent changes in synaptic protein complex composition are consistent in different detergents despite differential solubility
Source: Sci Rep. 2019 Jul 26;9:10890. doi: 10.1038/s41598-019-46690-y (PMC6659712; doi:10.1038/s41598-019-46690-y)
Supplement: Supplementary file 1 — Supplementary Info [file 41598_2019_46690_MOESM1_ESM.pdf]

Supplementary Information for

Activity-dependent changes in synaptic protein complex composition are consistent in different detergents despite differential solubility

Authors: Jonathan D. Lautz[1], Edward P. Gniffke[1], Emily A. Brown[1,2], Karen B. Immendorf[1], Ryan D. Mendel[1], Stephen E.P. Smith[1,2,3*]

Figures S1 and S2

Table S1: Lysis buffer composition

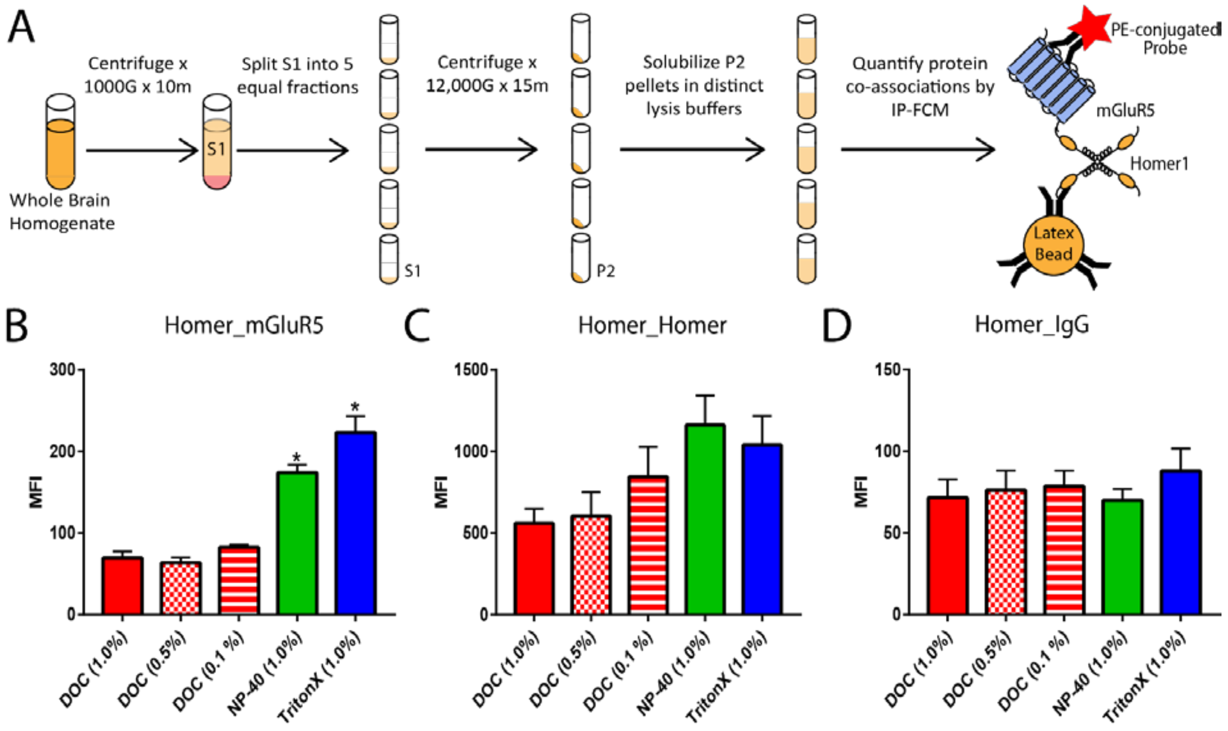

Figure S1: (A) Workflow. (B-D) Quantification of Homer_mGluR5, Homer_Homer and Homer_IgG in five lysis buffers. N=3 experiments. *p <0.05, paired Student's t-test.

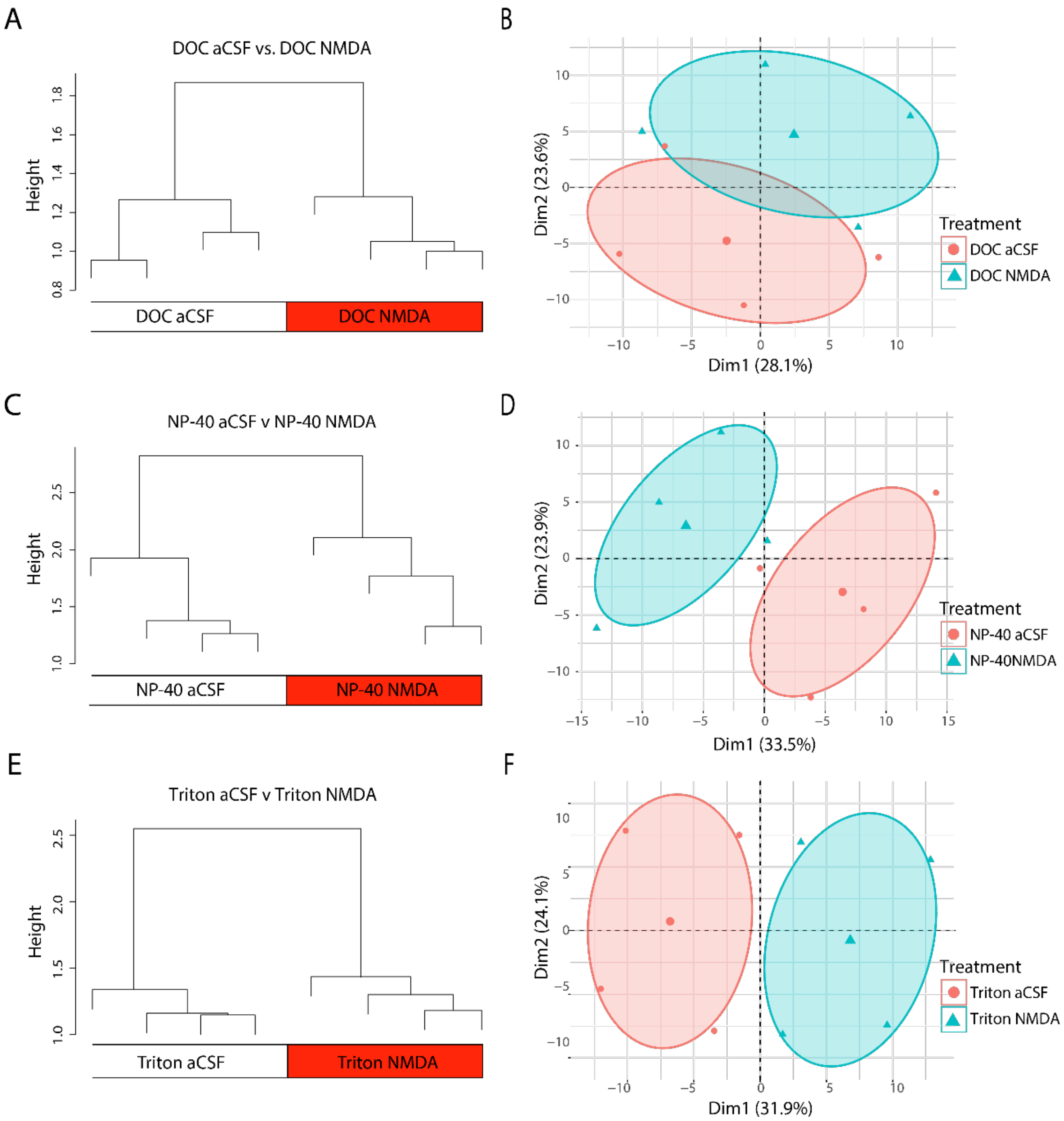

Figure S2: PCA and Clustering of NMDA stimulation analyzed separately for each detergent. A,B) Clustering and PCA for NMDA vs. aCSF for DOC. C,D) Clustering and PCA for NMDA vs. aCSF for NM-40. E,F) Clustering and PCA for NMDA vs. aCSF for Triton.

Table S1

Lysis Buffer	Recipe
DOC (pH 9.0)	50 mM Tris (pH 9.0), 30 mM NaF, 5 mM sodium orthovanadate + Protease/phosphatase inhibitor cocktails (Sigma), .02 mM $ZnCl_2$
DOC (TBS)	150mM NaCl, 50mM Tris (pH 7.4), DOC, 10 mM NaF, 2 mM sodium orthovanadate + Protease/phosphatase inhibitor cocktails (Sigma)
NP-40 (TBS)	150mM NaCl, 50mM Tris (pH 7.4), 1% NP-40, 10 mM NaF, 2 mM sodium orthovanadate + Protease/phosphatase inhibitor cocktails (Sigma)
Triton (TBS)	150mM NaCl, 50mM Tris (pH 7.4), 1% Triton, 10 mM NaF, 2 mM sodium orthovanadate + Protease/phosphatase inhibitor cocktails (Sigma)
DOC (EGTA)	150mM NaCl, 50mM Tris (pH 7.4), 1% DOC, 10 mM NaF, 2 mM sodium orthovanadate + Protease/phosphatase inhibitor cocktails (Sigma), 2 mM EGTA
NP-40 (EGTA)	150mM NaCl, 50mM Tris (pH 7.4), 1%NP-40, 10 mM NaF, 2 mM sodium orthovanadate + Protease/phosphatase inhibitor cocktails (Sigma), 2 mM EGTA
Triton (EGTA)	150mM NaCl, 50mM Tris (pH 7.4), 1% Triton, 10 mM NaF, 2 mM sodium orthovanadate + Protease/phosphatase inhibitor cocktails (Sigma), 2 mM EGTA
DOC (HEPES)	150mM NaCl, 50mM HEPES (pH 7.4), DOC, 10 mM NaF, 2 mM sodium orthovanadate + Protease/phosphatase inhibitor cocktails (Sigma)
NP-40 (HEPES)	150mM NaCl, 50mM HEPES (pH 7.4), NP-40, 10 mM NaF, 2 mM sodium orthovanadate + Protease/phosphatase inhibitor cocktails (Sigma)
Triton (HEPEPS	150mM NaCl, 50mM HEPES (pH 7.4), Triton, 10 mM NaF, 2 mM sodium orthovanadate + Protease/phosphatase inhibitor cocktails (Sigma)
